# Sex/Gender Differences in Brain Health Pathways Linking Education to Cognitive Trajectories

**DOI:** 10.1093/geroni/igaf122.1514

**Published:** 2025-12-31

**Authors:** Diefei Chen, C Shaaban, Ileana De Anda-Duran, Dahyun Yi, Shannon Risacher, Paul Crane, A Kraal

**Affiliations:** Johns Hopkins Bloomberg School of Public Health, Baltimore, Maryland, United States; University of Pittsburgh, Pittsburgh, Pennsylvania, United States; Tulane University, New Orleans, Louisiana, United States; Seoul National University, Seoul, Republic of Korea; Indiana University School of Medicine, Indianapolis, Indiana, United States; University of Washington, Seattle, Washington, United States; Columbia University, New York, New York, United States

## Abstract

Educational attainment may protect against poor cognitive health outcomes in later-life potentially via cerebrovascular and neurodegeneration pathways implicated in Alzheimer’s disease (AD). Underlying brain health mechanisms may vary by sex/gender and ethnicity, given group differences in educational opportunities. This study aims to characterize sex/gender differences in brain health pathways linking education to later-life cognition in Korean and US research study cohorts. Participants comprised older adults without dementia from Korea (KBASE: N = 434, age=70±8, education=11±5, 57% women) and the US (ADNI: N = 375, age=71±7, education=17±2, 53% women; all non-Latinx white). Latent growth models tested sex/gender differences in the indirect effects of education on four-year cognitive trajectories via markers of cerebrovascular disease (white matter hyperintensities (WMH)) and neurodegeneration (hippocampal volume and cortical thickness of AD-signature regions), stratified by sex/gender and cohort, adjusting for age, APOE genotype, intracranial volume, and cardiometabolic conditions. Among women in KBASE, lower WMH burden mediated the associations of higher education with better executive function and memory levels and slower executive function decline. Larger hippocampal volume mediated the associations of higher education with better memory and executive function levels and slower memory decline. Among women in ADNI, hippocampal volume mediated the association between education and memory level, but not memory change. Mediation was not observed in men in KBASE and ADNI. Cortical thickness did not mediate associations. Findings suggest potential sex/gender differences in neurobiological pathways linking education to cognition in older adulthood. Future research is needed to characterize sociocultural factors that may shape educational opportunities and brain health across diverse populations.

